# Percutaneous Compression Plate versus Dynamic Hip Screw for Treatment of Intertrochanteric Hip Fractures: A Meta-Analyse of Five Randomized Controlled Trials

**DOI:** 10.1155/2014/512512

**Published:** 2014-03-10

**Authors:** Lei Zhang, Jie Shen, Shengpeng Yu, Qiang Huang, Zhao Xie

**Affiliations:** ^1^National & Regional United Engineering Laboratory of Tissue Engineering, Department of Orthopaedics, Southwest Hospital, Third Military Medical University, Chongqing 400038, China; ^2^Department of Orthopaedics, The 118th Hospital of PLA, No. 15 Jiafusi Xiang, Wenzhou, Zhejiang 325000, China

## Abstract

*Background*. Percutaneous compression plating (PCCP) has been advocated to reduce blood loss, relieve pain, and lead to faster rehabilitation for the treatment of intertrochanteric fractures. The purpose of this meta-analysis was to estimate the outcomes and complications of the PCCP versus dynamic hip screw (DHS) fixation for intertrochanteric fractures. *Methods*. All randomized controlled trials (RCT) that compared PCCP with DHS in treating adult patients with intertrochanteric fractures were included. Main outcomes were collected and analysed using the RevMan 5.1 version. *Results*. Five trials met the inclusion criteria. Compared with DHS, PCCP had similar operation time (95% CI: −26.01~4.05, *P* = 0.15), length of hospitalization (95% CI: −1.79~1.25, *P* = 0.73), mortality (95% CI: 0.37~1.02, *P* = 0.06), incidence of implant-related complications (95% CI: 0.29~1.82, *P* = 0.49), and reoperation rate (95% CI: 0.41~3.05, *P* = 0.83). But blood loss (95% CI: −173.84~−4.81, *P* = 0.04) and transfusion need (95% CI: −0.53~−0.07, *P* = 0.01) significantly favored the PCCP. *Conclusions*. The PCCP was associated with reduced blood loss and less transfusion need, but similar to DHS in other respects. Owing to the limitations of this systematic review, more high-quality RCTs are still needed to assess the clinical efficiency of PCCP.

## 1. Introduction

Proximal femoral Fractures are a major source of morbidity and mortality in today's ageing population [1, 2]. Ninety percent of hip fracture patients are over the age of 65, and the majority of them are combined with age-related diseases [3], approximately 20% of the patients died within 1 year [4]; the mortality is increasing [5]. Moreover, the number of hip fractures is rising more rapidly than can be accounted for by demographic changes alone [6]. Approximately 50% of hip fractures occur in the intertrochanteric region [7]. To return to preinjury function and activity levels, early operative interventions have gradually become the preferred solution for the treatment of senile femoral intertrochanteric fracture [8].

The DHS and its variants serves had been considered the standard implant in the treatment of intertrochanteric hip fractures [9] with a high cost performance for stable intertrochanteric fracture [10]. However, for unstable intertrochanteric fractures, the failure rate is higher [11–13]. Currently, although there are many choices of internal fixation device, DHS is still the most frequently used implant for treating these fractures [14]. But this surgical procedure can be associated with substantial blood loss, soft-tissue damage, and worsening of existing comorbidities in the elderly patient [3].

In the late 1990s, Gotfried developed the PCCP technique, which minimises operative trauma by way of two small percutaneous portals, and small-diameter drilling prevents additional bone damage in the remaining lateral trochanteric wall [15–17]. Some biomechanical studies have demonstrated that PCCP, as a double-axis neck screw configuration with a sliding capability, provides rotational stability and controlled fracture impaction, prevents collapse, and resists angular displacement [18–21]. Therefore, Panesar et al. proposed that PCCP would become the “gold standard” in the treatment of intertrochanteric hip fractures [22]. Which is the better for intertrochanteric hip fracture either PCCP or DHS? Several prospective randomised controlled trials (RCT) spanning 10 years suggest that PCCP can reduce intraoperative blood loss, shorten operation time, and relieve postoperative pain [23–26], even if there is no significant difference in device-related complications, mortality, and functional recovery compared with DHS [23, 26, 27]. On the contrary, other reports suggest that PCCP lengthens operation time [27], increases biomechanical complications [24], reduces the incidence of fracture collapse [25, 28], and better improves the functional recovery [29].

Meta-analysis is a statistical method used within a systematic review, which can integrate the results of different independent studies addressing the same question into a quantitative summary [30, 31], increase the statistical power and precision of estimates for defined endpoints, and draw robust conclusions from conflicting reports. Therefore, meta-analyses and systematic reviews are often advocated as the best source of evidence to guide both clinical decision and healthcare policy [32, 33]. Recently, systematic review and meta-analysis were conducted to test the theoretical advantages of PCCP fixation over DHS fixation for intertrochanteric hip fractures by Cheng et al. [34] and Ma et al. [35]. But there were some quasirandomised controlled trials (qRCT) and nonrandomized controlled trials (NRCT) in the inclusion literatures, which reduced the confidence level of meta-analysis. No well-designed meta-analysis and systematic review capable of providing high levels of evidence has been conducted. So, we carry out this study and use meta-analytical techniques to evaluate clinical outcomes and safety of PCCP from RCTs compared with DHS.

## 2. Methods

This study was performed in line with recommendations from the Cochrane Collaboration and the Preferred Reporting Items for Systematic Reviews and Meta-Analyses statement [36].

### 2.1. Search Strategy

Literatures were identified through a computerized search in Medline, Embase, and the Cochrane Controlled Trials Register using the following terms: PCCP, percutaneous compression plate, extracapsular hip fracture, intertrochanteric fracture, and peritrochanteric fracture from January 1998 to August 2013. Specific retrieval style was not listed here.

### 2.2. Inclusion and Exclusion Criteria

Included studies had to fulfill the following inclusion criteria: (1) comparison of PCCP with DHS techniques in patients treated for intertrochanteric hip fractures, (2) research methods of the studies were limited in prospective randomized controlled trials, (3) patients older than 18 years, (4) the articles were restricted to English language, and (5) when two studies were reported by the same institution and authors, either the one of the higher quality or the most recent publication was included in the analysis, unless the study outcomes were mutually exclusive or measured at different time intervals.

Exclusion criteria were (1) type of literature as a “review” and “digest,” “talk,” “letters,” “commentary,” and “case report” and (2) qRCT or NRCT, especially the articles published in recent years.

Articles obtained from the electronic search were screened by two independent reviewers (Lei Zhang and Jie Shen) based on the inclusion criteria and exclusion criteria. Disagreements between the reviewers were resolved by discussion to reach a consensus.

### 2.3. Data Extraction and Quality Assessment

Two of authors (Lei Zhang and Jie Shen) independently extracted the following data from the qualifying articles. The data extracted from the studies included the title, first author, time, geographical, study design, patient characteristics (including age, gender, number of patients, side of fracture, fracture classification, and follow-up), and patient-based outcomes measures. The corresponding author of each study was contacted to obtain any missing information that was required. The extracted data were rechecked by an additional author (Zhao Xie). Literature quality assessment was performed by two independent researchers (Lei Zhang and Jie Shen). Disagreement was resolved by discussion. A third researcher (Zhao Xie) was the adjudicator, when no consensus was achieved. The focus of quality assessment was risk of bias in all included studies. And the evaluation criteria and methods follow the Cochrane Collaboration's proposal and use their tool RevMan5.1 for assessing risk of bias [37].

### 2.4. Data Synthesis and Analysis

We performed data synthesis and analysis with the Review Manager software provided by Cochrane Collaboration (RevMan Version 5.1; The Nordic Cochrane Center, The Cochrane Collaboration, Copenhagen, Denmark). The assessment for statistics heterogeneity was calculated through Chi^2^ and *I* ^2^ test. When the Chi^2^ was *P* > 0.05 or *I* ^2^ < 20% indicating low statistical heterogeneity, a fixed effect model was used. A random effect model was used when Chi^2^ was *P* < 0.05 and *I* ^2^ > 20%. For continuous data, means and standard deviations were pooled to a weighted mean difference (WMD) and 95% confidence interval (CI) in the meta-analysis. For binary data, odds ratio (OR) and 95% confidence interval (CI) were assessed (*α* = 0.05 for the inspection standards) [37]. Publication bias was assessed by using a funnel plot of the most frequently reported outcome.

## 3. Results

**Figure 1:**
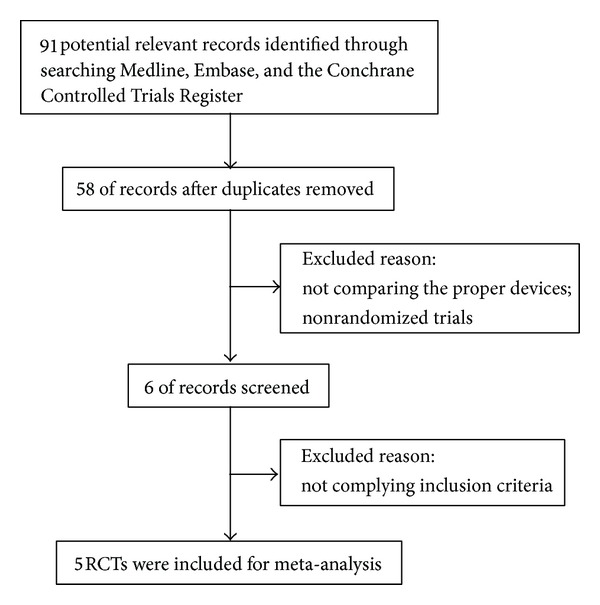
Study flow diagram.

A total of 91 articles were initially identified through a computerized search. After reading the titles and abstracts, 33 duplicates were removed. According to the inclusion and exclusion criteria, 6 RCT studies comparing PCCP with DHS eventually satisfied the inclusion criteria in the remaining 58 papers. Then, manually search for these articles and read the full texts. An article in Spanish was finally excluded due to a lack of randomization method [38]. Two studies [23, 24], performed in the same center, had different inclusion criteria for type of fractures. Therefore, these two studies were all included. At last, 5 RCT studies were included [23–27]. Study flow diagram was listed in Figure 1.

**Figure 2:**
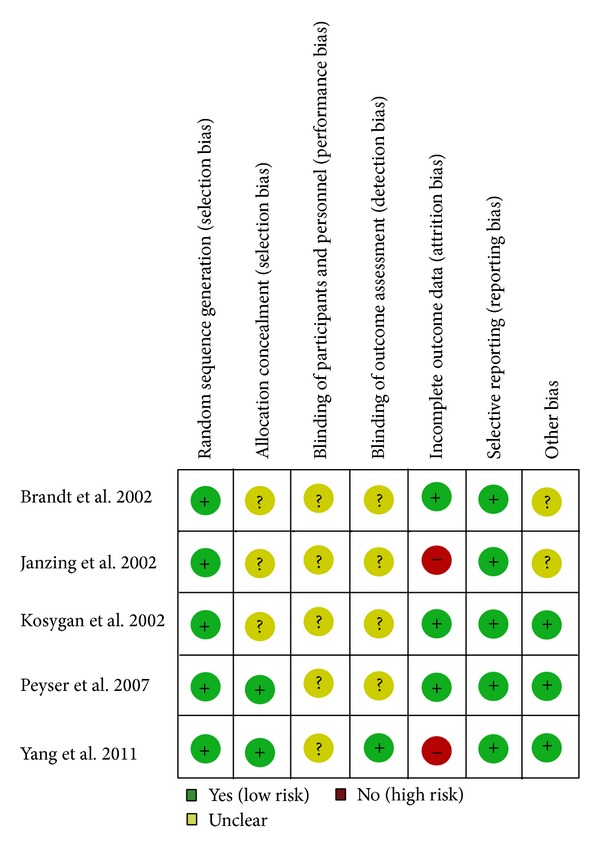
Methodological quality of included studies. This risk of bias tool incorporates assessment of randomization (sequence generation and allocation concealment), blinding (participants, personnel, and outcome assessors), completeness of outcome data, selection of outcomes reported, and other sources of bias. The items were scored with “yes,” “no,” and “unclear.”

Among the 5 included RCT studies, all reported adequate generation of allocation sequence, but only two trials [25, 26] reported allocation concealment. Whilst surgeon blinding was inappropriate in this study design, and four studies did not blind their assessors to patients group. Only one trial [26] performed the single blinding to assessors. Two studies [24, 26] had patients lost to follow-up. Three studies [25–27] reported that they did not receive any grant in support of their research. The methodological quality of included studies was presented in Figure 2.

The demographic characteristics of studies included are summarized in Table 1. Five RCTs involving 463 patients were eligible for inclusion, with individual sample size ranging from 66 to 115 patients. Two hundred and twenty-one patients were treated with PCCP and 242 with DHS. There were two studies undertaken in Belgium, one, respectively, in USA, Israel, and England.

### 3.1. Operation Time

**Figure 3:**
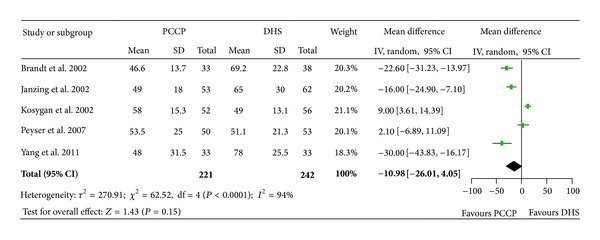
Comparison of operation time between PCCP and DHS.

All Five studies [23–27] provided data of operation time and were eligible in the form of mean and standard deviation (SD). There were 463 fractures included, 221 patients with the PCCP fixation and 242 with the DHS fixation. The heterogeneity test indicated a statistical evidence of heterogeneity (Chi^2^ = 62.52, *P* < 0.00001, *I* ^2^ = 94%). We pooled data by a random effect model which indicated that there was no statistical difference in operation time between the two groups. (Mean difference: −10.98, 95% CI: −26.01~4.05, *P* = 0.15) (Figure 3).

### 3.2. Blood Loss and Transfusion

**Figure 4:**
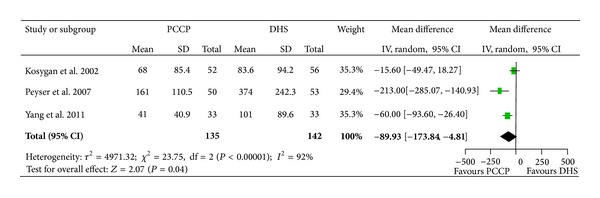
Comparison of blood loss between PCCP and DHS.

**Figure 5:**
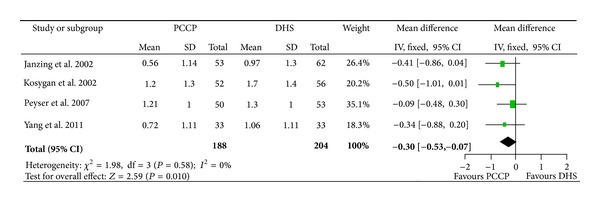
Comparison of transfusion units per person between PCCP and DHS.

There were three articles [25–27] involving 177 fractures which provided data of blood loss. The heterogeneity test indicated that there was a statistical heterogeneity (Chi^2^ = 23.75, *P* < 0.00001, *I* ^2^ = 92%), and data pooled by a random effect model indicated that the blood loss significantly favored the PCCP (mean difference: −89.32, 95% CI: −173.84~−4.81, *P* = 0.04) (Figure 4). There were four articles [24–27] included with 392 fractures providing data for blood transfusion. The heterogeneity test indicated no statistical heterogeneity (Chi^2^ = 1.98, *P* = 0.58, *I* ^2^ = 0%), and data pooled by a fixed effect model indicated that transfusion units per person significantly favored the PCCP (mean difference: −0.30, 95% CI: −0.53~−0.07, *P* = 0.010) (Figure 5).

### 3.3. Length of Hospitalization

**Figure 6:**
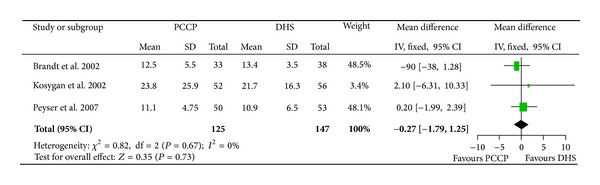
Comparison of length of hospitalization between PCCP and DHS.

Three studies [23–27] included data of hospital stay. There were a total of 282 patients, with 135 patients in the PCCP group and 147 in the DHS group. The heterogeneity test indicated no statistical heterogeneity (Chi^2^ = 0.82, *P* = 0.67, *I* ^2^ = 0%). Data pooled by a fixed effects model indicated that there was no statistical difference in hospital stay between the PCCP group and DHS group (mean difference: −0.27, 95% CI: −1.79~1.25, *P* = 0.73) (Figure 6).

### 3.4. Implant-Related Complications

**Figure 7:**
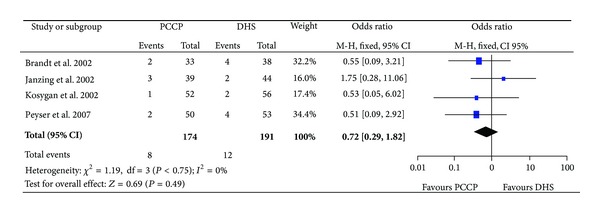
Comparison of implant-related complications between PCCP and DHS.

Four articles [23–25, 27] provided data of implant-related complications, which mainly included cut-out and perforation of femoral head [23–25, 27], protrusion of neck screw [23, 24, 27], osteonecrosis of the femoral head [25], varus collapse [25], fracture of the lateral cortex of the femur [24, 25], breakage of the implant [23], and redisplacement of the fractures [25]. The heterogeneity test indicated no statistical heterogeneity (Chi^2^ = 1.19, *P* = 0.75, *I* ^2^ = 0%), and data pooled by a fixed effect model indicated that there was no statistical difference in hospital stay between the PCCP group and DHS group. (OR: 0.72, 95% CI: 0.29~1.82, *P* = 0.49) (Figure 7).

### 3.5. Reoperation

**Figure 8:**
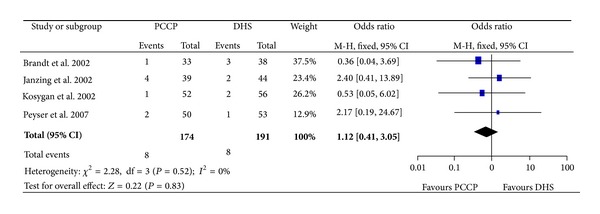
Comparison of reoperation between PCCP and DHS.

Four articles [23–25, 27] provided data of reoperation. The heterogeneity test indicated no statistical heterogeneity (Chi^2^ = 2.28, *P* = 0.52, *I* ^2^ = 0%), and data pooled by a fixed effect model indicated that the outcome of reoperation was similar between the PCCP group and the DHS group (OR: 1.12, 95% CI: 0.41~3.05, *P* = 0.83) (Figure 8).

### 3.6. Mortality

**Figure 9:**
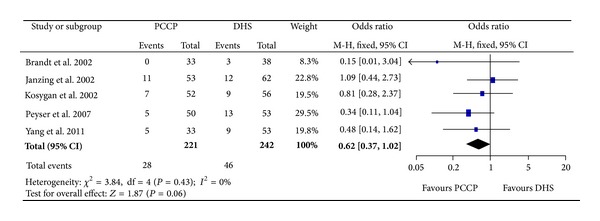
Comparison of mortality between PCCP and DHS.

All five studies [23–27] provided data of mortality. There were 463 fractures included, 221 patients with the PCCP fixation and 242 with the DHS fixation. The heterogeneity test indicated no statistical evidence of heterogeneity (Chi^2^ = 3.84, *P* = 0.43, *I* ^2^ = 0%) and data pooled by a fixed effect model indicated no statistical significant difference between the two groups (OR: 0.62, 95% CI: 0.37~1.02, *P* = 0.06) (Figure 9).

### 3.7. Complications

There were no statistically significant differences between PCCP and DHS for complications such as wound infection rates, respiratory, pulmonary embolism, cerebrovascular accident, cardiovascular events, and DVT (Table 2).

### 3.8. Publication Bias

**Figure 10:**
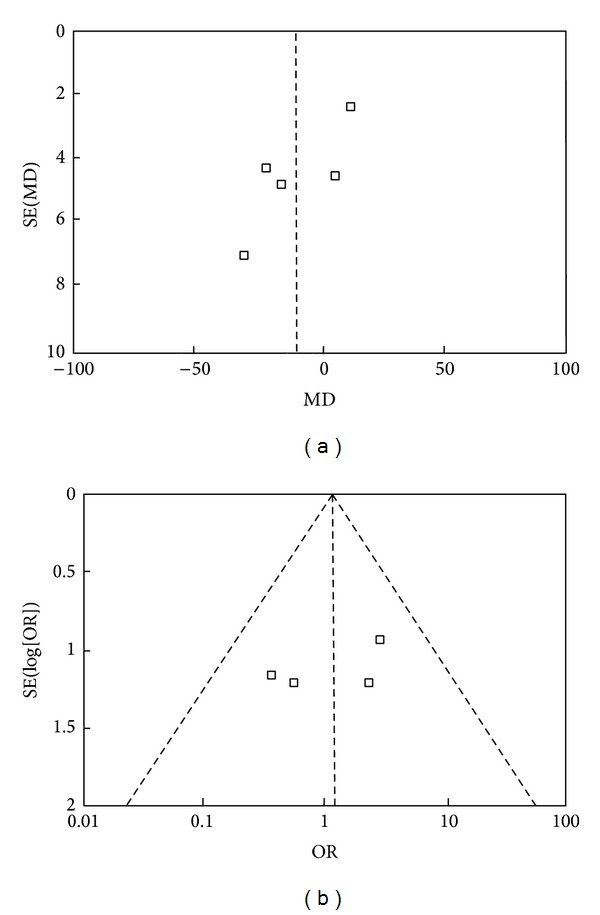
Funnel plot for operation time and the rate of reoperation demonstrating the evidence of publication bias. Basic symmetry of the above two funnel plot suggested that the possibility of publication bias is small ((a) operation time and (b) reoperation).

The assessment of publication bias using frequency of reoperation indicated a mild asymmetry. The funnel plot of operation time and the rate of reoperation demonstrated limited evidence of small study exclusion and publication bias with an asymmetrical diagram with few studies plotted on the right side of the funnel (Figure 10).

## 4. Discussion

With the rapid increase in the elderly population, the morbidity of intertrochanteric femoral fractures is also displaying a rising trend [39]. The treatment of intertrochanteric femoral fractures remains a challenge for orthopaedic surgeons because there is no unified standard in fracture classification, treatment, and curative effect evaluation. Although the techniques and devices have been continuously improved, the best choice of internal fixation method has been a focus of dispute. This is due to the complexity of the fracture itself and the onset in older patients. Intertrochanteric femoral fractures are a complex coupling of fragile patients with fragile bone. Generally, the operation is successful in terms of healing of the fracture, but the patient is unable to regain the preinjury level of function and independence. Promotion of fracture healing at the same time as regaining the preinjury level of function as much as possible had become the key goals of hip fracture treatment.

Minimally invasive surgery has gained in popularity in modern orthopaedic trauma because it has been shown to be associated with reduced operation time, decreased bleeding and postoperative pain, reduced postoperative morbidity, and faster recovery of function. The purpose of this meta-analysis and systematic review was to summarize the existing high levels of evidence to determine the safety and efficacy of the minimally invasive technique PCCP compared with DHS. This study demonstrated that PCCP can reduce blood loss and transfusion need compared with DHS for treating intertrochanteric fracture. However, no significant differences were observed between the two groups in terms of operation time, length of hospitalization, mortality, implant-related complications, and reoperation.

The short operation time is extremely important for elderly patients with multimorbidity, as the other diseases may take precedence and rapid fracture fixation is necessary, which can keep the influence of anaesthesia on respiration and blood circulation to a minimum. But, in our meta-analysis, there were no significant differences between the two groups in terms of operation time. Kosygan et al. [27] confirmed that the operation time was slightly longer with PCCP compared with DHS. Surgical learning curve is an important factor that may have accounted for the differences. Kosygan et al. [27] found that the operation time, particularly during the learning period, is longer in the PCCP group than that in the DHS group and believed that learning curve is more demanding for PCCP when compared with the relative simplicity of the DHS. On the contrary, some studies [23, 40, 41] have reported a shorter operative time and almost no learning curve with this new device when placed by both senior surgeons and residents.

The blood loss and transfusion need in PCCP were reduced significantly compared with DHS, which could be attributed to the percutaneous technique, less soft-tissue damage, and minimally operative trauma without exposing the fracture. Less blood loss can reduce transfusion need and avoid the hazards of blood transfusion, which is considering the rising costs, disease transmission, risk of transfusion reactions, and immunomodulation [42–44] for elderly patients with multimorbidity. In contrast, Peyser et al. [25] reported that the number of blood transfusion was inconsistent with blood loss during operation, since the principle blood loss occurred from the fracture site itself and not from the surgical exposure. Additionally, the perioperative hemoglobin level and the different indications for blood transfusion determined the rates or the units of transfusion, the latter of which was of high relevance to the hospital of included trials and study design.

There was no significant difference between PCCP and DHS in terms of the hospital stay. Because length of hospitalization is more depending on prevailing medical/economic/social conditions, five included RCTs were performed in four different countries, which have different medical environment and health care system. These are all decided the length of hospitalization. So, it is difficult to compare these results between studies.

Some biomechanical studies have demonstrated that PCCP, as a double-axis neck screw configuration with a sliding capability, provides rotational stability and controlled fracture impaction, prevents collapse, and resists angular displacement [18–21]. But, in terms of rate of implant-related complications and reoperation in this meta-analysis, the PCCP system has theoretical biomechanical advantages rather than practical advantages for the treatment of hip fractures compared with the DHS system. Surgical learning curve is still an important factor. Schmidt-Rohlfing et al. [45] indicated that the surgeon-related risk factors (number of operations, operation time, and TAD) seem to be more relevant for the reoperation rate after internal fixation with the PCCP device when compared with the patient-related risk factors and a substantial learning curve is more demanding for PCCP. If surgeons are unfamiliar with the new implant, a number of operative complications and a conversion to an open procedure may occur. This phenomenon is more often in early RCTs studies [23, 24, 27]. Yang et al. [46] found that this surgery can be performed well with a short learning curve. After recognition of the two major technical pitfalls (insufficient reduction and malposition of the lower neck screw), further technical problems could hypothetically be avoided.

Mortality of PCCP has a decreased trend compared with DHS; the reduction in blood loss and surgical trauma in the PCCP group may significantly reduce postoperative morbidity and facilitate recovery in elderly patient, but the difference was not significant (*P* = 0.06). Peyser et al. [40] suggested that reduction in overall complications and cardiovascular complications did not affect the mortality in both groups. Kenzora et al. [47] showed a high correlation between mortality rate and preexisting medical conditions. Endo et al. [48] confirmed that men were more likely to sustain a medical complication and had a higher mortality at 1 year compared to women. Besides gender, Kannegaard et al. [49] found that higher age and multimorbidity to be related to an increased risk of dying within the first year after fracture and acute complications might be one of the explanations. So, it is important to emphasise the particular rigorous postoperative diagnostic evaluation and treatment of comorbid conditions in the male hip fracture patient.

Complications rates were statistically similar in both PCCP and DHS group. The most common and hazardous complications after hip fracture surgery are cardiac and pulmonary complications [50]. Peyser et al. [40] reported that reduced surgical trauma and decreased bleeding are thought to be possible explanations for the reduced cardiovascular complications with PCCP. Ma et al. [35] reported that there was a statistically significant difference in respect to cardiovascular events between PCCP and DHS. In our analysis, three trials reported complications [23, 25, 27], but Brandt's [23] reported that data was general, so two trials' data [25, 27] was included to make statistical analysis (Table 2). There was a discrepancy in the complications between our study and Ma et al. [35] Furthermore, Matot et al. [51] found a correlation between fewer cardiovascular events and reduced pain in elder patients with proximal femoral fracture. Although postoperative pain was not assessed in our meta-analysis, Janzing et al. [24] reported that minimal invasive treatment of pertrochanteric fractures with the PCCP reduces operation time and postoperative pain.

With the aging of the population, intertrochanteric hip fracture is associated with increased morbidity. Patients with intertrochanteric fractures imposed serious health and economic burden to the society. The PCCP technique can reduce blood loss and transfusion need, which can reduce costs. In addition, a trend toward a shorter surgical time or anesthesia time, lower mortality, and reduced incidence of implant-related complications and reoperation was observed in the PCCP group. Incorporating formal health economic analysis in the future randomized control trials would be useful for providing greater clarity in decision making.

Our study focus on systematic reviews of RCTs, which are likely to provide more reliable information than other sources of evidence on the differential effects of alternative forms of healthcare [52]. Undoubtedly, this analysis also has some limitations. First, our systematic reviews focus on RCTs in English, and, due to the small number of RCTs, the patient number included in our study was limited. Furthermore, inclusion criteria were restricted, which can lead to selection or allocation biases, affecting the results of the meta-analysis. Second, insufficient data were available to support the meta-analysis of pain scores, functional outcome scores, or quality of life outcome measures for different time point and scoring scale; it is difficult to make significant statistical conclusion in terms of function recovery.

## 5. Conclusions

Based on this systematic review and meta-analysis, PCCP has no obvious statistical difference in terms of operation time, hospital stay, mortality rate, reoperation rate, systematic complications, and device-related complications compared with DHS. However, PCCP results in decreased blood loss and reduced transfusion requirement, while maintaining at least equivalent functional outcomes and success rates in fracture fixation. Thus, PCCP system is recommended as a minimally invasive technique that can be considered as an additional alternative treatment for intertrochanteric fractures, especially in elderly patients with multimorbidities. Future well-designed RCTs are needed to determine whether PCCP is associated with reduced operation time and better functional recovery compared with DHS.

## Figures and Tables

**Table 1:** The characteristics of included studies.

Study	Year	Country	Study design	Number of Patients		Mean age (range)			Gender (M/F)			Side of fracture (R/L)		Fracture classification	Follow-up (month)	Conflicts of interests
				PCCP	DHS	PCCP	DHS	*P *	PCCP	DHS	*P *	PCCP	DHS			
Yang et al. [26]	2011	USA	RCT	33	33	76 (30–100)	77 (44–101)	>0.05	11/22	8/25	>0.05	N/S	N/S	AO/OTA 31.A1-A2	36	No
Peyser et al. [25]	2007	Israel	RCT	50	53	78.9 (62–95)	82.4 (63–95)	0.04	16/34	18/35	>0.05	27/23	32/21	AO/OTA 31.A1-A2	12	No
Kosygan et al. [27]	2002	England	RCT	52	56	82.7 (53–93)	82.8 (57–97)	>0.05	8/44	12/44	>0.05	N/S	N/S	Evans1A-D	6	No
Brandt et al. [23]	2002	Belgium	RCT	33	38	80.1 (63–96)	81.6 (61–97)	>0.05	N/S	N/S	>0.05	16/17	12/26	Evans1A-D	3	N/S
Janzing et al. [24]	2002	Belgium	RCT	53	62	82 (65–96)	83 (64–98)	>0.05	N/S	N/S	>0.05	N/S	N/S	AO/OTA 31.A1-A2	12	N/S

PCCP: percutaneous compression plate; DHS: dynamic hip screw; RCT: randomised controlled trial; M: males; F: females; N/S: not stated; R: right; L: left.

**Table 2:** Meta-analysis results of complications comparing PCCP with DHS.

Complications	Group (*n*)		Studies (*n*)	Overall effect		
	PCCP	DHS		Effect estimate	95% CI	*P* value
Respiratory	102	109	2	0.70	0.28~1.75	0.45
Pulmonary embolism	102	109	2	0.52	0.09~2.93	0.46
Cardiovascular event	102	109	2	0.80	0.33~1.92	0.61
Cerebrovascular accident	102	109	2	0.17	0.02~1.42	0.10
DVT	102	109	2	0.33	0.08~1.44	0.14
Superficial wound infection	102	109	2	0.35	0.04~3.41	0.37

DVT: deep-vein thrombosis; PCCP: percutaneous compression plate; DHS: dynamic hip screw; CI: confidence interval.
